# The Many Faces of Immune Activation in HIV-1 Infection: A Multifactorial Interconnection

**DOI:** 10.3390/biomedicines11010159

**Published:** 2023-01-08

**Authors:** Laura Mazzuti, Ombretta Turriziani, Ivano Mezzaroma

**Affiliations:** 1 Department of Clinical and Molecular Medicine, Sapienza University of Rome, 00185 Rome, Italy; 2 Laboratory of Virology, Department of Molecular Medicine, Sapienza University of Rome, 00185 Rome, Italy; 3 Department of Translational and Precision Medicine, Sapienza University of Rome, 00185 Rome, Italy

**Keywords:** HIV-1, AIDS, inflammaging, immune activation, microbial translocation, pro-inflammatory cytokines

## Abstract

Chronic immune activation has a significant role in HIV-1 disease pathogenesis and CD4+ T-cell depletion. The causes of chronic inflammation and immune activation are incompletely understood, but they are likely multifactorial in nature, involving both direct and indirect stimuli. Possible explanations include microbial translocation, coinfection, and continued presence of competent replicating virus. In fact, long-term viral suppression treatments are unable to normalize elevated markers of systemic immune activation. Furthermore, high levels of pro-inflammatory cytokines increase susceptibility to premature aging of the immune system. The phenomenon of “inflammaging” has begun to be evident in the last decades, as a consequence of increased life expectancy due to the introduction of cART. Quality of life and survival have improved substantially; however, PLWH are predisposed to chronic inflammatory conditions leading to age-associated diseases, such as inflammatory bowel disease, neurocognitive disorders, cardiovascular diseases, metabolic syndrome, bone abnormalities, and non-HIV-associated cancers. Several approaches have been studied in numerous uncontrolled and/or randomized clinical trials with the aim of reducing immune activation/inflammatory status in PLWH, none of which have achieved consistent results.

## 1. Introduction

Systemic chronic immune activation and CD4+ T-cell depletion characterize the progression of human immunodeficiency virus type-1 (HIV-1) infection toward acquired immune deficiency syndrome (AIDS). However, the causal link between these two phenomena has not been formally established. Persistent activation is observed in numerous components of both the innate and adaptative immune system, including cells (e.g., activated phenotypes of macrophages and dendritic cells), cytokines and chemokines [tumor necrosis factor, interleukin (IL)-1, IL-6, IL-8, IL-15, and IL-10], acute phase proteins (serum amyloid A, C-reactive protein), elements of the coagulation cascade (D-dimers, tissue factor), elements of fibrosis (matrix metalloproteinase activation, collagen deposition), and microbial sensors (lipopolysaccharide binding protein, soluble CD14). Increased turnover and exhaustion of T cells and turnover of B cells are observed with an altered phenotypic profile and hypergammaglobulinemia [1]. Moreover, high levels of systemic immune activation and inflammation not only promote viral replication and CD4+ T-cell apoptosis, but they may also lead to a more rapid decline of immune function and competence, which have been associated with aging [2].

With the introduction of combined antiretroviral therapy (cART), the immune responses, morbidity, and mortality of people living with HIV (PLWH) have significantly improved. The life expectancy of PLWH has dramatically increased and is only slightly shorter than that of uninfected individuals, so the effects of aging on HIV-1-positive patients have begun to be evident [3]. Several disorders that typically affect the aged population now appear in relatively young HIV-1 subjects, such as neurocognitive disorders, cardiovascular diseases (CVD), metabolic syndrome (MS), bone abnormalities, and non-HIV-1-associated cancers [4,5]. The Strategies for Management of Antiretroviral Therapy (SMART) study demonstrated that non-AIDS-defining age-related comorbidities are the major cause of morbidity and mortality, compared to opportunistic diseases [6]. Most of these pathologies have been linked to immune-senescence and inflammageing, a type of premature aging present in PLWH.

However, the availability of long-term viral suppression treatment has not been successful in normalizing elevated markers of systemic immune activation [7]. Even today, the mechanisms underlying the establishment and maintenance of chronic immune activation are unclear. This incomplete understanding of AIDS pathogenesis is one of the causes of the absence of a vaccine or eradication strategy [8].

## 2. Role of Immune Activation in Progression to AIDS

Studies of pathogenic and nonpathogenic models of simian immunodeficiency virus (SIV) infection have provided insight into the role of systemic immune activation in the progression to AIDS. Natural SIV hosts, such as African green monkeys (AGMs) and sooty mangabeys (SMs), generally are able to live normally with the virus and do not progress to immunodeficiency, despite sustained high levels of plasma viremia. On the contrary, in other non-human primates, such as rhesus macaques (RMs) and Asian pigtailed macaques, SIV infection results in immunodeficiency and progression to AIDS, similar to HIV-1 infection [9]. During both pathogenic and non-pathogenic infection, robust viral replication and early antiviral responses occur during the acute phase of infection, but they show interesting immunological differences: SMs do not exhibit the increased CD4+ T-cell turnover and generalized immune activation that is characteristic of SIV infection in RMs or HIV-1 infection in humans [10,11,12]. Natural hosts have evolved strategies to avoid disease progression and achieve an effective response, which enables symbiotic coexistence. The observed adaptations include early resolution of acute T-cell activation, rather than improved viral control. Natural hosts have the ability to attenuate acute innate immune responses to SIV after a few weeks of infection, contrary to pathogenic SIV and HIV-1 infections in which immune activation persists throughout the course of the disease. SIV-infected SMs have low levels of immune activation, T-cell turnover, and cell cycle perturbation compared to SIV-infected RMs or HIV-1-infected humans, with more comparable levels to uninfected animals [13,14,15,16]. Moreover, non-pathogenic infections do not reveal microbial translocation, as shown by lack of LPS or sCD14 in the plasma of SIV- infected RMs or AGMs [17,18], and experimentally induced immune activation with LPS in natural hosts shows significantly increased virus replication and CD4+ T-cell depletion [19]. These observations about the lack of chronic immune activation and microbial translocation in disease progression observed in natural hosts have brought attention to the role of immune activation in HIV-1 infection. The precise mechanisms underlying the resolution of acute immune activation in SIV-infected SMs and AGMs remain poorly understood and are likely quite complex.

Another observation supporting the role of immune activation as the major driving force of CD4+ T-cell loss and AIDS is provided by the elite controller group (ECs). ECs represent a small subset of PLWH (about 3/1000) [20,21,22] who are able to maintain a stable CD4+ T-cell count (irrespective of a threshold) and have a viral load persistently below 50 copies/mL for more than 12 months [23], even in the absence of cART. It appears that viral genetic defects or humoral responses in ECs do not play a major role in controlling HIV-1 viral load, whereas innate responses of potential interest have been reported [24]. Interestingly, AIDS events have been described in a few ECs with loss of CD4+ T cells, despite maintaining undetectable viral loads [25,26,27]. The study of progression to AIDS in ECs has provided invaluable insight for deeper comprehension of immune mechanisms controlling HIV-1 infection and disease progression. Indeed ECs, despite spontaneously viral replication control, show higher levels of T-cell activation than healthy donors and even higher levels than cART-suppressed individuals [28]. These individuals with higher T-cell activation display slow but progressive CD4+ T-cell loss and can develop AIDS, emphasizing the role of immune activation in the pathogenesis of HIV-1 infection.

## 3. Proposed Mechanisms Inducing Chronic Immune Activation

**Figure 1:**
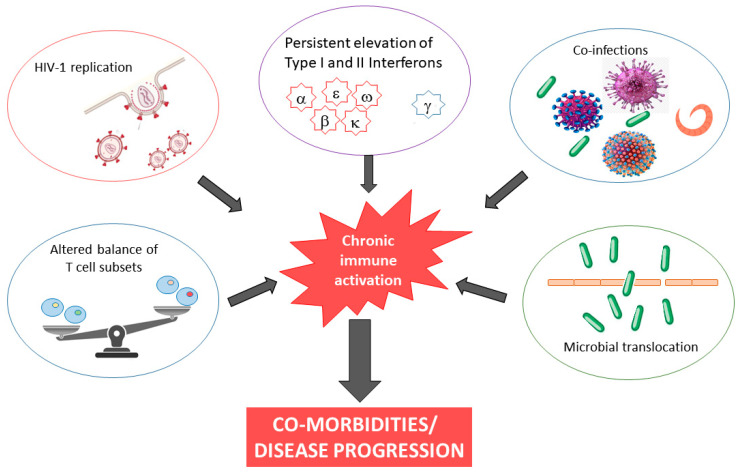
Causes and consequences of chronic immune activation in HIV-1 infection.

The causes of chronic inflammation and immune activation in HIV-1 infection are incompletely understood but are likely multifactorial in nature, involving both direct and indirect stimuli. Although our understanding remains incomplete, possible explanations include microbial translocation, co-infection, and continued presence of HIV RNA, almost always present at levels below the detection limits of clinical assays, in cART-treated subjects (Figure 1). Finally, HIV-1 infection is characterized by persistent elevation of type I and II interferons. It has been demonstrated that inadequate regulation of IFN responses drives chronic immune activation [29,30].

The most obvious cause of immune activation is direct antigenic stimulation by the virus and its products, such as Nef and gp120, which stimulate the activation of lymphocytes and macrophages, resulting in the secretion of pro-inflammatory cytokines and chemokines. The viral protein Nef, for example, has been shown to reduce endothelial nitric oxide production, promote secretion of endothelial-cell derived MCP-1, induce endothelial cell apoptosis, and increase inflammatory cytokine release from macrophages [31,32]. In addition, HIV-1 components also bind to pattern recognition receptors, such as Toll-like receptors (TLR) 7 and 9 [33,34]. Del Cornò and colleagues provided evidence of an interplay between HIV-1 gp120 and host TLR4 in human monocyte-derived macrophages and hepatic stellate cells, which led to intracellular pathways and biologic activities that mediate proinflammatory and profibrogenic signals. In particular, this interaction resulted in the activation of the NF-kB and MAPK pathways, leading to downstream up-regulation of pro-inflammatory cytokines and chemokines in human monocyte-derived macrophages and to cell migration and secretion of CCL2 and CXCL8 in hematopoietic stem cells [35].

The level of viremia also correlates with the level of immune activation, as shown in cART-treated subjects or ECs. It has been estimated that only approximately 20% of circulating CD8+ T cells are HIV-1 specific in untreated chronically infected patients [36], whereas HIV-1-specific CD4+ T cells are usually present at a lower magnitude, up to approximately 3% of circulating CD4+ T cells [37]. However, high levels of HIV-1 replication are insufficient to induce pathological levels of immune activation. Furthermore, recent data report that inflammation is not directly associated with the size of the blood reservoir, neither with total HIV DNA [38] nor with intact proviral DNA levels [39,40,41]. Interestingly, a strong positive correlation between HIV DNA and CD8+ T-cell activation was found in viremic patients with primary or chronic infection, whereas there was no correlation was found between T-cell activation and HIV DNA in patients with successfully treated chronic infection [42].

Latent viruses, such as cytomegalovirus (CMV) and Epstein-Barr virus (EBV), reactivate more frequently during HIV-1 infection due to the depletion of CD4+ T cells and loss of CD8+ T cells that control viral replication. This reactivation contributes to the continued stimulation of the immune system [43,44,45]. Moreover, other viruses can also contribute to immune activation, such as hepatitis B or C viruses (HBV or HCV). In fact, different levels of inflammation exist between patients co-infected with HIV-1 and HBV or HCV compared with mono-infected and uninfected controls. Several studies have shown that HIV/HCV and HIV/HBV co-infected patients have higher levels of plasma inflammation and microbial translocation biomarkers than HIV-1 mono-infected patients and these markers were positively correlated with indices of hepatic damage [46,47,48]. In addition to such chronic viral infections, different pathogens can cause systemic immune activation and dysregulation of the immune system to a similar extent as HIV-1 infection. For example, non-HIV-associated immune activation observed in chronic helminthic infections could contribute to CD4+ T-cell loss and dysregulated immune response in PLWH co-infected with helminths, even if a specific role in disease progression has not been sufficiently demonstrated [49].

The massive depletion of CD4+ T cells in the gastrointestinal tract (GUT) during primary infection and the concomitant accumulation of inflammatory cells, such as dendritic cells, neutrophils, and monocytes, progressively compromise the mucosal integrity and disrupt the mucosal barrier [50,51]. This dysregulation favors microbial translocation, resulting in translocation of peptidoglycan, lipoteichoic acid, LPS, flagellin, and CpG DNA from the intestinal lumen into the systemic circulation. Stimulating several TLRs, the microbial products active a signaling cascade and induce the production of pro-inflammatory cytokines, such as IL-1β, IL-6, TNF-α, and type I interferons [52]. This local and systemic inflammation caused by microbial translocation contributes to aberrant immune activation in chronic HIV-1 infection.

Moreover, several studies reported that sCD14 plasma levels in PLWH were significantly higher than in HIV-1-negative subjects, but were similar among PLWH stratified according to plasma viral load, even in those with residual viral replication [53,54,55].

## 4. The Detrimental Consequences of Systemic Immune Activation

The persistent state of immune activation and inflammation in HIV-1 infection has extensive and detrimental effects on the host immune system and patient outcome. Immune system dysregulation, characterized by a shift in leukocyte activity and an imbalance in cytokine levels, plays a pathogenic role in the setting of HIV-1 infection. Since the virus preferentially infects and kills activated CD4+ T-helper cells [56], the repertoire of these cells is altered and the loss of T-cell homeostasis compromises the host’s ability to control a wide range of potential pathogens. The contemporaneous presence of high levels of CD4+ T-cell activation and high levels of viremia leads to further new infection and consequent death of CD4+ cells. Moreover, the depletion of CD4+ T cells triggers a homeostatic response of the immune system, which stimulates the activation and proliferation of surviving cells to replenish the compartment, providing further targets for the virus. This dysregulation is exacerbated by the inhibition of normal function in B-cells, NKs, and other antigen-presenting cells, as well as by the achievement of persistent replicative senescence in T cells [57,58]. In addition, elevated levels of HIV-1-associated immune activation products, such as pro-inflammatory and pro-apoptosis cytokines, sustain generalized damage to the host immune system. Indeed, many studies report uncommon levels of many cytokines, such as pro-inflammatory IL-1β, IL-2, IL-6, IL-8, and TNF-α, but also anti-inflammatory cytokines, such as IL-4, IL-10, and IL-13. Moreover, increased levels of MIP-1α, ICAM, VCAM, MCP-1, and CXCL9 were found [59,60,61,62,63,64].

**Figure 2:**
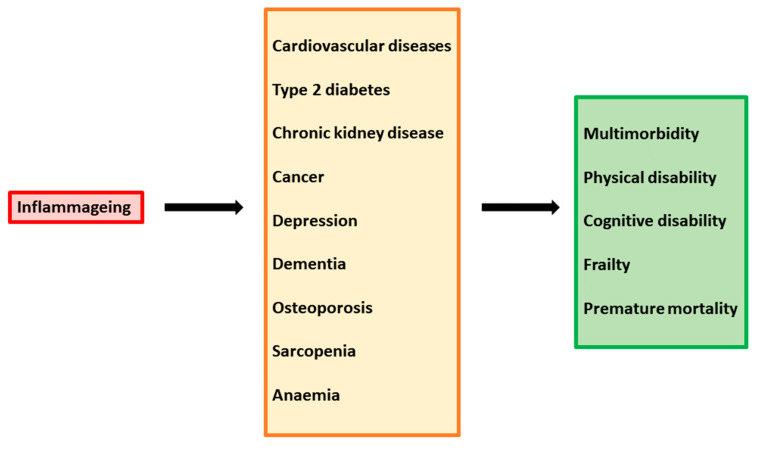
Inflammaging as a driver of multiple chronic diseases.

Immune system dysregulation also results in inflammatory damage to the architecture of tissue involved in T-cell regeneration and function, such as bone marrow, the thymus, and lymph nodes. In particularly, altered thymic function results in suboptimal production of naïve T cells, greater differentiation of naïve cells into effector/memory cells, and hindered immune reconstitution [65,66,67]. Importantly, the chronic stimulation of the immune system and high levels of pro-inflammatory cytokines increase susceptibility to premature aging of the immune system. The phenomenon of “inflammageing” has begun to be evident in the last decades, as a consequence of increased life expectancy due to the introduction of cART. In cART-treated patients, quality of life and survival have improved substantially; however, these individuals are predisposed to chronic inflammatory conditions leading age-associated diseases, such as inflammatory bowel disease, neurocognitive disorders, cardiovascular diseases, metabolic syndrome, bone abnormalities, and non-HIV-associated cancers (Figure 2).

During the chronic phase of HIV-1 infection, both the accelerated process of immune senescence and inflammaging may contribute to the development of the progressive immunodeficiency [68].

## 5. Effect of cART on HIV-1 Associated Immune Activation

Although the introduction of cART has made it possible to achieve durable control of viral replication, cART is not curative and cannot eradicate HIV-1 from the body. Antiretroviral drugs prevent the capacity of HIV-1 to replicate, which can be defined as the spread of infectious virus from one cell to another cell. These drugs do not target integrated HIV DNA and are unable to eliminate long-lived cells that harbor proviruses; thus, despite cART being fully suppressive, the virus will persist for decades. During cART, viral replication is effectively controlled in many PLWH and HIV-1 viral loads are suppressed to below detectable levels. However, if treatment is stopped, HIV-1 usually rebounds to high levels [69]. Even when cART-treated subjects have undetectable viral loads based on current clinical assays, ultrasensitive methods can still reveal HIV RNA in plasma [70]. The source of this persistent, low-level viremia remains unclear; it is probably derived from ongoing rounds of viral replication, or activation of infected resting T cells in the latent reservoir, or some combination of the two [71]. Although it is theoretically possible that residual viremia stimulates the immune system and contributes to CVD, these relationships have yet to be proven [72].

Iannetta and colleagues reported that cART was able to reduce myeloid and lymphoid inflammation in advanced and non-advanced PLWH by increasing circulating plasmacytoid cell counts and normalizing HLA-DR expression on myeloid dendritic cells and non-classical monocytes, even during the first year of treatment [73].

In cART-treated PLWH with undetected viremia, the level of inflammation markers is dramatically reduced compared to baseline; however, cART has not been successful in normalizing elevated markers of systemic immune activation [74]. For example, levels of several pro-inflammatory molecules, such as CRP, IL-6, and D-dimer [75], as well as markers of T-cell activation [76,77], remain higher in PLWH than in uninfected controls despite suppressive cART, and this increase has been associated with higher mortality [78,79,80,81,82]. Early initiation of cART, within the first 6 months of infection, seems to achieve lower levels of immune activation than when treatment is started even after a few years [83]. However, initiation of cART during acute HIV-1 infection is insufficient to resolve the chronic inflammation, and the inflammation in these patients remains higher than in uninfected controls even when cART is started early.

In cART-treated patients, levels of pro-inflammatory cytokines are also associated with increased risk of CVD, independently from other CVD risk factors [84,85], and with infection-related and unrelated cancers, even after adjusting for demographics and CD4+ T cell counts [86]. In addition, higher levels of TNF-α were also found to be significantly associated with the occurrence of serious non-AIDS events [87].

## 6. Gender Differences in HIV-1 Associated Immune Activation

In recent years, some studies have shown that sex is implicated in HIV-1 pathogenesis. Biological sex is an important contributor to disease pathogenesis in multiple infectious diseases [88], with a distinct genetic complement, hormonal environment, and behavioral and social context. Sex differences have been described for diverse aspects of HIV-1 infection and disease, including transmission, pathogenesis, morbidity, mortality, and response to antiretroviral treatment. In addition, sex difference seems to influence immune activation and HIV-1-associated co-morbidities [89]. Furthermore, sex-specific differences in CD4+ T cell counts have been reported in several studies, both in naïve and in cART-treated patients: females had higher CD4 cell counts and fewer AIDS-defining illnesses [90,91,92]. Moreover, it has been demonstrated that chronically HIV-1-infected women have significantly higher levels of CD8+ T-cell activation than men with the same HIV-1 viral load, thereby experiencing a significantly increased risk of developing AIDS compared to men with similar levels of HIV-1 replication [93]. Systemic immune activation markers have been shown to be higher in HIV-1 infected women than in HIV-1 infected men or uninfected women [94]. Higher expression of IFN-stimulated genes was observed in women. In the short term, a hyper-acute innate immune response to infection could allow women to better control viral replication; however, with time, this hyper-stimulation of the immune system could lead to a dysfunctional response.

To date, the precise mechanisms responsible for these reported sex differences in viral load, T cell count, and immunological differences remain unknown. This elevated immune-activation in women could result from sex-specific environmental risk factors, sex differences in the microbiome [95], steroid hormones secreted by gonads [96], and direct effects of X and Y chromosome-linked factors [97]. Several genes on the X chromosome can potentially influence immunocompetence; in particular, the X chromosome encodes for FOXP3, the lineage-defining transcription factor of regulatory T cells; IL-2Rγ, a common cytokine receptor; and pattern recognition receptors (PRR) TLR7 and TLR8, which are known to sense HIV-1 ssRNA. Notably, X chromosome inactivation is a random process and an estimated 20% of the X chromosome escapes inactivation that, consequently, may lead to overexpression of certain gene products [98]. In addition, growing evidence supports a potential epigenetic regulation of sex differences in immune responses [99]. Other studies suggest that sex hormones influence HIV-1 acquisition through changes in microbiome composition, for example, reducing bacterial vaginosis could modulate the gut microbiome and thus contribute to systemic inflammation [100]. Sex hormones are also involved in IFN-α production since estrogen and progesterone have been reported to modulate plasmacytoid dendritic cell secretion [101,102].

## 7. Experimental and Clinical Approaches to Decrease Chronic Inflammation and Immune Activation

Several immunosuppressive drugs were studied in numerous uncontrolled and/or randomized trials in HIV-1 infected patients, none of which achieved solid results.

Prednisolone use in naïve subjects showed a decrease in activation markers with increased HIV RNA levels, but no effects in cART-treated patients were reported and, more importantly, no significant effect was determined on the primary endpoint of HIV-1 disease progression to AIDS [103,104,105,106].

Cyclosporin A (CsA) and rapamycin (RAPA), two immunosuppressive agents used both in autoimmune disorders as well as for prophylaxis or treatment of rejection following organ transplantation, were tried in different kinds of PLWH. These agents showed several effects on the immune system, acting on different populations of T lymphocytes, including CD4+ T cells, and interfering with the secretion of many cytokines. Moreover, in vitro experiments with RAPA and CsA showed a suppressive effect on HIV-1 reactivation with decreased production of cytokines such as IL-2, MCP-1, MIP-1α, IL-1β, IFN-γ, TNF-α, and IL-6 [107,108,109,110]. Patients with acute infection treated with cART and CsA exhibited an increased number of CD4+ T cells compared with those treated with cART alone. In vivo use of RAPA following liver transplantation in PLWH showed suppressive activity on HIV-1 replication but no effects on CD4+ T cell counts [111].

Agents with immunosuppressive properties such as chloroquine/hydroxychloroquine (CQ/HCQ), widely used in treating autoimmune conditions, were shown to suppress HIV-1 replication in patients [112]. Several studies with different doses of these drugs were performed in PLWH, achieving contrasting results. In some trials, adding CQ/HCQ to cART reduced immune activation markers on CD8+ T cells or levels of activated CD4+/Ki67+ and CD14+/CD69+ T cells [113], whereas HCQ did not reduce CD8+ T-cell activation and IL-6/D-dimer levels in other trials, with an increase of viral replication and a decline in CD4+ T cell count [114,115]. These conflicting results could be partially caused by different doses of CQ/HCQ utilized in the trials.

Due to the well-known anti-inflammatory properties of statins reducing the release of pro-inflammatory cytokines at the vessel level, these anti-cholesterol agents were also used in several trials in PLWH with cART, aimed at reducing inflammation and immune activation at different stages of viral infection. A reduction in circulating activated CD4+ and CD8+ T cell subsets (i.e., HLA-DR+ and CD38+ expressing cells) was observed, whereas no statistically significant changes were reported for levels of pro-inflammatory cytokines [116,117,118].

Lowering bacterial translocation may have a promising impact on HIV-1 disease progression. Different studies in PLWH showed that improving the microbiota composition and reducing mucosal and systemic inflammation with probiotic/prebiotic administration was able to decrease microbial translocation and immune activation [119,120,121]. However, no beneficial effects were observed in other studies [122,123]; thus, clear evidence supporting this approach remains insufficient.

Some hypoglycemic agents are able to modify the microbiota composition, promote gut barrier integrity, and reduce inflammation in human and animal models of diabetes. Taking these properties into account, metformin and sitagliptin were used with cART in PLWH affected by metabolic syndrome or impaired glucose tolerance, showing the ability to reduce inflammation and chronic immune activation by different mechanisms [124,125,126,127,128].

Other anti-inflammatory drugs have been tested to reduce chronic inflammation and immune activation in PLWH, also aimed at decreasing cardiovascular events associated with persistent infection. Aspirin, clopidogrel, and COX-2 inhibitors were used in several studies showing mixed results both on activation marker expression and soluble markers of inflammation [129,130,131,132].

Other molecules, such as cannabis, pyridostigmine, dipyridamole, mesalazine, and leflunomide, which are able to exert anti-inflammatory effects or decrease immune activation in different settings, are under investigation in PLWH, but the results have been inconsistent to date [133,134,135,136,137,138].

## 8. Conclusions

As previously described, several mechanisms are involved in HIV-1-related immune activation and inflammation. To date, the persistence of HIV-1 viral reservoirs, disruption of the intestinal barrier with associated microbial translocation, depletion of regulatory T (Treg) cells, and coinfection with other viruses have all been proposed as drivers of persistent immune activation. Gender also seems to affect the inflammation status of HIV-infected patients. Moreover, elevated levels of biomarkers representative of these factors are associated with dramatic increases in the risk of progressive disease and non-AIDS-related pathologies, including all-cause mortality. Levels of IL-6, sCD14 and sCD163 (both released by monocytes/macrophages), non-specific markers of inflammation (such as C-reactive protein and cystatin C), and markers of hypercoagulation and microbial translocation are variably increased during HIV-1 infection. It has also been demonstrated that levels of activated T cells remain elevated during chronic cART, despite suppressed HIV-1 replication, and appear to be related to the size of the HIV-1 reservoir. Furthermore, the rate at which CD4+ T cell counts increase during ART also has prognostic significance. A variable proportion of individuals fails to achieve a CD4+ T cell number above 500/μL, despite chronic suppression of HIV-1 replication, and this suboptimal recovery increases the risk of many comorbidities (heart, renal, metabolic, bone diseases, and cancers) as well as all-cause mortality. Again, a persistently elevated CD8+ T cell count, which leads to partial restoration of the CD4/CD8 ratio, has an important prognostic significance on clinical outcomes, immune dysfunction, and HIV-1 reservoir size in long-term treated patients. A low CD4/CD8 ratio is a predictor of mortality both in the aging general population and HIV-1-infected individuals. Patients with CD4+ T cell recovery and a low CD4/CD8 ratio have increased immune activation and a higher risk of non-AIDS morbidity and mortality. In accordance with these observations, the CD4/CD8 ratio may be a useful tool in the immunological evaluation of ART-treated patients.

Nevertheless, the study of all these markers of inflammation/immune activation and viral reservoir persistence, while having made a huge contribution to the understanding of their role in the development of co-morbidities, all-cause mortality, and the pathogenesis of HIV-1 disease, has not demonstrated a substantial contribution to the clinical management of patients. In fact, none of these markers has risen to the role of an element to be evaluated in the initial staging of patients and none are included in the currently available guidelines for the management and therapeutic follow-up of HIV-1-infected subjects.

Only the CD4/CD8 ratio and number of CD8+ T cells are currently indicated in the EACS guidelines [139] as markers to be evaluated at baseline and during follow-up of patients under ART, as numerous studies on large case series have demonstrated their role as predictive markers of immune reconstitution and normalization of immune functions. In fact, in addition to the absolute number and percentage of CD4+ T cells, they represent strong predictors of the risk of non-AIDS events and mortality in HIV-1 patients undergoing antiretroviral therapy.

As previously discussed, from the perspective of reducing inflammation and immune dysfunction, several drugs with non-specific immunomodulating effects and other anti-inflammatory drugs (i.e., statins, chloroquine, hydroxychloroquine, COX-2 inhibitors, aspirin, and methotrexate) have been used in prospective interventional trials as possible adjuncts to standard antiretroviral drugs. However, studies with these molecules have shown inconsistent or contradictory results, so none of them have been actually indicated in addition to antiretroviral therapies.

In conclusion, despite progress in understanding the phenomena underlying aberrant inflammation and immune activation in HIV-1 infection, an effective clinical intervention strategy remains lacking. Starting cART at an early stage of HIV-1 infection, before the viral reservoir is established, may be a promising approach. Hope for a cure for HIV-1 infection is anticipated in the results of several studies now implementing eradication strategies.

## Data Availability

Not applicable.
